# Telomerase regulation by the long non-coding RNA H19 in human acute promyelocytic leukemia cells

**DOI:** 10.1186/s12943-018-0835-8

**Published:** 2018-04-27

**Authors:** Joëlle El Hajj, Eric Nguyen, Qingyuan Liu, Claire Bouyer, Eric Adriaenssens, George Hilal, Evelyne Ségal-Bendirdjian

**Affiliations:** 1 INSERM UMR-S 1007, Cellular Homeostasis and Cancer, Paris, France; 2 Paris-Descartes University, Paris Sorbonne Cité, Paris, France; 3 Paris-Sud University, Paris-Saclay University, Orsay, France; 4 Cancer and Metabolism Laboratory, Faculty of Medicine, Saint-Joseph University, Beirut, Lebanon; 5 INSERM U 908, University Lille 1, Villeneuve d’Ascq, France; 6 Present address: Bristol-Myers Squibb (China) Investment Co. Ltd., Shanghai, 200040 People’s Republic of China; 7 INSERM UMR-S 1007, Paris-Descartes University, 45 rue des Saints-Pères, 75006 Paris, France

**Keywords:** Telomerase, hTERT, *hTR*, *H19* long non-coding RNA, Retinoids, Acute promyelocytic leukemia

## Abstract

**Background:**

Since tumor growth requires reactivation of telomerase (hTERT), this enzyme is a challenging target for drug development. Therefore, it is of great interest to identify telomerase expression and activity regulators. Retinoids are well-known inducers of granulocytic maturation associated with *hTERT* repression in acute promyelocytic leukemia (APL) blasts. In a maturation-resistant APL cell line, we have previously identified a new pathway of retinoid-induced *hTERT* transcriptional repression independent of differentiation. Furthermore, we reported the isolation of a cell variant resistant to this repression. Those cell lines could serve as unique tools to identify new telomerase regulators.

**Methods:**

Using a microarray approach we identified the long non-coding RNA, *H19* as a potential candidate playing a role in telomerase regulation. Expression of *H19, hTERT*, and *hTR* were examined by quantitative reverse transcriptase polymerase chain reaction (qRT-PCR). Telomerase activity was quantified by quantitative telomeric repeats amplification protocol (qTRAP). In vitro and in vivo assays were performed to investigate *H19* function on telomerase expression and activity.

**Results:**

We showed both in retinoid-treated cell lines and in APL patient cells an inverse relationship between the expression of *H19* and the expression and activity of hTERT. Exploring the mechanistic link between *H19* and hTERT regulation, we showed that *H19* is able to impede telomerase function by disruption of the hTERT-*hTR* interaction.

**Conclusions:**

This study identifies a new way of telomerase regulation through *H19*’s involvement and thereby reveals a new function for this long non-coding RNA that can be targeted for therapeutic purpose.

**Electronic supplementary material:**

The online version of this article (10.1186/s12943-018-0835-8) contains supplementary material, which is available to authorized users.

## Background

Human telomerase is a special ribonucleoprotein enzyme that stabilizes chromosome ends by adding (TTAGGG)_n_ telomeric sequences and thus has a key role in maintaining telomere length and in cellular replicative life-span. This ribonucleoprotein, usually absent or expressed at a low level in most normal somatic cells, is highly active in cancer cells, and plays a key role in cell immortalization and tumorigenesis [1, 2]. Due to this differential expression pattern, telomerase has been proposed as a promising target for anticancer therapies. Therefore, different therapeutic approaches for telomerase-based treatment of cancer have been developed [3, 4]. The main levels on which telomerase activity can be targeted are associated with transcription of *hTERT* and *hTR* genes, as well as disruption of the telomerase complex assembly, inhibition of the assembled telomerase complex and its interaction with telomeres [4].

Retinoids are well-known inducers of granulocytic maturation of primary acute promyelocytic leukemia (APL) blasts. Previous studies, including our own on the NB4 cellular model of APL, showed that *hTERT* repression is associated with cell differentiation. In a maturation-resistant APL cell line (NB4-LR1), we showed that retinoids can regulate telomerase and telomere length independently of cell maturation leading to growth arrest and cell death [5, 6]. Moreover, we reported the isolation of a variant of the NB4-LR1 cell line, named NB4-LR1^SFD^, which is resistant to ATRA-induced cell death. In NB4-LR1^SFD^ cells, hTERT has been stably reactivated despite the continuous presence of ATRA [7]. This stable telomerase reactivation after an initial step of downregulation seems similar to what occurs during tumorigenesis when telomerase becomes reactivated. Therefore, the NB4-LR1^SFD^ cell line is a valuable cell model to study the molecular events occurring during the oncogenic reactivation of telomerase.

Using a microarray approach to identify genes differentially modulated by ATRA treatment in NB4-LR1 and NB4-LR1^SFD^ cells, we found an inverse correlation between the expression of hTERT and the long non-coding RNA, *H19*. We set out to explore this potential correlation and the underlying mechanistic link between *H19* expression and hTERT regulation and showed that *H19* is able to impede telomerase function by disrupting the hTERT-*hTR* interaction. This finding identifies for the first time a new way of telomerase regulation by retinoids through *H19*’s involvement and thereby reveals a new function of this long non-coding RNA.

## Methods

### Chemicals, cell lines, and cell cultures

All-*trans* retinoic acid (ATRA), 8-(4-chlorophenylthio)adenosine 3′,5′-cyclic adenosine monophosphate (8-CPT-cAMP), and protease inhibitor cocktail (P8340) were purchased from Sigma (St Louis, MO, USA). The maturation sensitive NB4 cells and both maturation-resistant human APL cell lines, NB4-LR1 and NB4-LR1^SFD^, were cultured as previously described [5]. The NB4-LR1^SFD^ cell line was isolated as a population of cells emerging from a culture of NB4-LR1 cells under the selective presence of ATRA (1 μM). It bypasses the death step induced by long-term ATRA treatment because of the reactivation of hTERT. The established NB4-LR1^SFD^ cell line is stable and able to grow either in the presence or in the absence of ATRA. This property of resistance to ATRA-induced cell death during a prolonged treatment is maintained for more than 6 months of culture in the absence of ATRA. Therefore both NB4-LR1 and NB4-LR1^SFD^ cells were routinely cultured in the same ATRA-free RPMI medium.

All cells were cultured at 37 °C in a humidified incubator with 5% CO_2_ (Binder Incubators, Nanterre, France). Cell density was determined every 2 or 3 days using Coulter counting, and when it reached 6-7 × 10^5^ cells/ml, cells were re-seeded in a new flask containing fresh medium.

### RNA extraction and processing for microarray experiments

NB4-LR1 and NB4-LR1^SFD^ cells were treated or not for 7 days with ATRA (1 μM). Total cellular RNA from 3 independent experiments (biological replicates) was extracted using TRIzol reagent (Invitrogen) according to the manufacturer’s instructions. After validation of the RNA quality with Bioanalyzer 2100 (using Agilent RNA6000 nano chip kit), 500 ng of total RNA was reverse transcribed following the GeneChip® WT Plus Reagent Kit (Affymetrix) instructions. Briefly, the resulting double strand cDNA is used for in vitro transcription with T7 RNA polymerase (all these steps are included in the WT cDNA synthesis and amplification kit of Affymetrix). After purification according to Affymetrix protocol, 5.5 μg of the cDNA obtained are fragmented and biotin-labeled using Terminal Transferase (using the WT terminal labeling kit of Affymetrix). cDNA is then hybridized to GeneChip™ Human Transcriptome Array 2.0 (HTA 2.0.) (Affymetrix) at 45 °C for 17 h.

After overnight hybridization, chips were washed on the fluidic station FS450 following specific protocols (Affymetrix) and scanned using the GCS3000 7G. The scanned images were then analyzed with Expression Console software (Affymetrix) to obtain raw data (CEL files) and metrics for Quality Controls. The observations of some of these metrics and the study of the distribution of raw data show no outlier experiments. CEL files were then normalized and processed to signal intensities using the RMA (robust multi-array average) algorithm from the Bioconductor library and the cdf file V20 from BrainArray. All subsequent analyses were done on the log (base 2) transformed data in Partek Genomics Suite: non supervised analysis and analysis of variance (ANOVA) were used to detect eventual outlier samples and to identify differentially expressed genes. A global view of the differentially expressed genes was drawn using a Volcano Plot, the log transformed adjusted *p*-values plotted on the y-axis and log2 fold change values on the x-axis. A cut-off value of *p* < 0.01 was chosen for statistical significance. The expression of the significant genes was displayed in a heatmap. The statistically significant differential gene expression list was further trimmed by considering only genes with │fold change│ > 2 as biologically significant. Venn diagrams were generated using Venny tool (http://bioinfogp.cnb.csic.es/tools/venny/index.html).

### Quantitative reverse transcriptase polymerase chain reaction (qRT-PCR)

Total RNA was isolated from cells as mentioned above. RNA concentration and quality were determined via A260/230 and A260/A280 nm absorbance with Nanodrop Spectrophotometer. One microgram of total RNA was subjected to reverse transcriptase (RT) reaction using Transcriptor First Strand cDNA Synthesis kit (Roche Diagnostics) according to the manufacturer’s instructions with random hexamer primers. The cDNAs were subsequently analyzed by quantitative real-time PCR using the LightCycler technology and the Light Cycler FastStart DNA MasterPLUS SYBR Green Kit (Roche Diagnostics) according to the manufacturer’s instructions. *hTERT*, *hTR* and *H19* levels were normalized to the expression of glyceraldehyde-3-phosphate dehydrogenase (*GAPDH*) serving as the internal control gene. Primer sequences are shown in Additional file 1: Table S1. All samples were analyzed in triplicate in at least three independent experiments.

### Quantitative RT- PCR for detection of miR-675-5p and miR-675-3p

Total RNA was extracted as previously described. The expression of miR-675-5p and miR-675-3p was quantified by quantitative RT-PCR. Single strand RNA is first polyadenylated by poly(A)polymerase before reverse transcription into cDNA using qScript RT with a proprietary adapter oligo(dT) primer. Using the kit: “the qScript microRNA cDNA Synthesis Kit” (Quanta Biosciences, VWR), following the manufacturer’s protocol. The step of amplification was carried out using the LightCycler technology, the PerfecCTa microRNA Assay, the PerfeCTa Universal PCR Primer and miRNA specific primers (Quanta Biosciences). SNORD44 serves as an internal control. The miRNA specific primers are listed in Additional file 1: Table S1.

### Production of RNA molecules for in vitro studies

Plasmids pcDNA 3.1(+)-H19 and pcDNA 3.1(+)-GFP (green fluorescent protein, used in control experiments) were linearized with the restriction enzyme BamH1 (20 U/μl). The products of digestion were purified using the plasmid DNA purification kit (Nucleobond® PC20, Macherey Nagel) and used as templates for subsequent in vitro transcription. *H19* and *GFP* RNA molecules were generated by in vitro transcription using the T7 RNA polymerase (Roche). The reaction was performed in a final volume of 20 μl containing 1 μg of linearized plasmid, 1× reaction buffer (40 mM Tris, pH 8.0, 6 mM MgCl_2_, 2 mM spermidine, 10 mM DTT, 10 mM each ATP, GTP, UTP, and CTP, 1 μl RNase inhibitor (20 U/μl), and 2 μl T7 RNA polymerase (20 U/μl). The reaction mix was incubated at 37 °C for 2 h before treatment with RNase-free DNase I (1 U/μl, Promega) at 37 °C for 30 min and the reaction was terminated by adding 2 μl 0.2 M EDTA and heating at 65 °C for 10 min. In vitro transcribed products were separated from unincorporated nucleotides with the RNeasy Mini kit (Qiagen) and stored at − 80° until used.

### Measurement of telomerase activity

Samples for telomerase activity assays were extracted following standard protocols. Briefly, cell pellets were resuspended (10^6^ cells in 200 μl) in lysis buffer (3-[(3-cholamidopropyl)-dimethylammonio]-1-propane-sulfonate, 10 mM Tris pH 8.0) and incubated on ice for 30 min. After centrifugation at 16000 g for 20 min. at 4 °C, aliquots of the supernatant were rapidly frozen and stored at − 80 °C. The protein concentration of extracts was determined with the BCA Protein Assay (Thermo Scientific).

Real-time quantitative telomeric repeat amplification protocol (qTRAP) assay was performed as described [8]. Briefly, reactions were carried out in 20-μl with LightCycler® FastStart DNA Master SYBR Green I (Roche) with 0.1 μg of telomerase primer TS, 0.05 μg of reverse primer ACX, and 2 μl of the sample to be tested. Samples were incubated 30 min. at 37 °C, 10 min. at 95 °C and amplified for 40 PCR cycles (5 s at 95 °C and 60 s at 60 °C). Data analysis was performed with the LightCycler software that integrates the real-time PCR efficiency that was calculated by serial dilution of the most active sample. Primer sequences are shown in Additional file 1: Table S1.

In vitro qTRAP was designed to test whether *H19* RNA fragments would inhibit telomerase enzymatic activity in vitro. Cell extracts (1 μg of protein) were mixed with the indicated amount of in vitro transcribed *H19* or *GFP* RNA and telomerase activity was quantified using the qTRAP assay as described above.

### Nucleofection of DNA constructs

Transfections were performed using the Amaxa Nucleofector technology. NB4 cells were transfected using 2 μg of either pcDNA 3.1 (+)-H19 or pcDNA 3.1 (+)-empty vector, using transfection solution V and program 001, according to the manufacturer’s instructions. Six hours after transfection, RNA and proteins were extracted as previously described. *H19* and *hTERT* levels were quantified by qRT-PCR, telomerase activity was measured using qTRAP assay as described above.

### Immunoprecipitation (IP) of hTERT-hTR complexes

To prepare the antibody-coated beads, 30 μl of protein A/G magnetic beads (Thermo Scientific) were incubated with 4 μg of polyclonal anti-hTERT antibodies or 4 μg of rabbit pre-immune IgG in 150 μl IP buffer (1% Igepal CA-630, 137 mM NaCl, 20 mM Tris-HCl at pH 8.0, 10% glycerol, and 2 mM EDTA) at 4 °C for at least 2 h. Then, the beads were washed three times with IP buffer and kept on ice until used. To prepare cell lysates, cells were harvested by centrifugation at 220 g and cell pellets re-suspended in 300 μl of freshly prepared lysis buffer (0.5% Na deoxycholate, 1% IGEPAL CA-630, 0.1% SDS, 150 mM NaCl, 50 mM Tris-HCl at pH 7.8, 10 mM EDTA, 1 × protease inhibitor cocktail, 1 mM PMSF, 5 mM NaF, 1 mM sodium orthovanadate, and 4 U/μl RNase inhibitor). The suspensions were incubated on ice for 20 min. After removing insoluble materials by centrifugation, lysates were transferred to tubes containing hTERT antibody (Rockland) or pre-immune IgG-coated beads, and IP was carried out by rotating the tubes at 4 °C overnight. Beads were then washed briefly three times and RNAs were eluted by incubating the beads in 100 μl buffer (20 mMTris-HCl pH 8.0, 137 mM NaCl, 10% glycerol, 1% NP-40, 2 mM EDTA) containing 0.8% SDS and 1.2 g/l Proteinase K, during 30 min. at 55 °C with shaking. Eluted beads were discarded using a magnet and the supernatant processed with TRIzol reagent. The resulting RNA preparations were analyzed for the presence of *hTR* by quantitative RT-PCR as described above.

In some experiments, 10 μg of in vitro transcribed *H19* RNA molecules were preincubated with 100 μg of cellular extracts for 30 min. at room temperature. This mixture was added to tubes containing hTERT antibody, and IP was carried out by rotating the tubes at room temperature for 90 min. The samples were then processed as above for *hTR* quantification. As a control, an in vitro transcribed *GFP* RNA was used.

### Statistical analysis

Statistical analysis was conducted using GraphPad Prism 6.01 software. The difference between groups was analyzed using unpaired or paired Student’s t-test when there were only two groups or assessed by one-way ANOVA followed by the Tukey’s multiple comparison tests when there were more than two groups. All tests carried out were two-tailed. Differences were considered as significant when *p* < 0.05.

## Results

### The induction of H19 expression by ATRA treatment of the maturation resistant NB4-LR1 and NB4-LR1^SFD^ cells is associated with hTERT repression

**Fig. 1:**
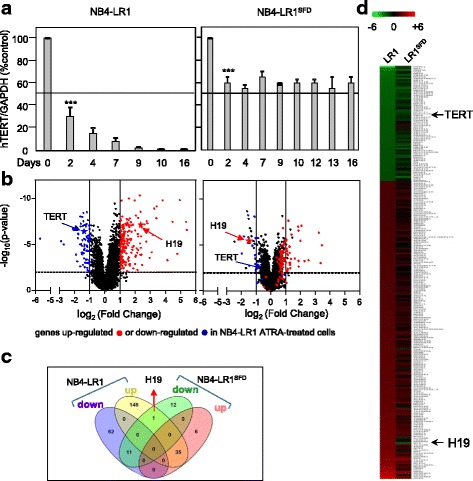
Microarray-based expression profiling. **a** Kinetics of hTERT expression in NB4-LR1 and NB4-LR1^SFD^ cells treated with ATRA (1 μM). **b** Volcano plot representation of the differentially expressed genes in a pair wise comparison of NB4-LR1 (left panel) and NB4-LR1^SFD^ (right panel) cells treated with ATRA (1 μM) for 7 days. The plot indicates -log_10_ (*p*-value) for genome-wide genes (Y-axis) plotted against their respective log_2_ (fold change) (X-axis). The significant cut-off was set to a *p*-value of 0.01 (−log_10_ (*p*-value) ≥ − 2, horizontal line), the biological cut-off was set to a fold change of ±2 fold (log_2_(fold change) < − 1 and > + 1, vertical lines). In ATRA-treated vs non-treated NB4-LR1 cells (left panel) biological and statistically insignificant genes are presented in black. A color was attributed to each gene whether it is significantly up-regulated (red) or down-regulated (blue). A star indicates *hTERT* and *H19* genes in blue and red, respectively. Importantly, in the NB4-LR1^SFD^ right panel, all genes retained the color defined in the NB4-LR1 left panel. Therefore *H19*, in red in the NB4-LR1 Volcano panel, remained in red in the NB4-LR1^SFD^ Volcano panel, even though its expression was repressed. **c** Heat map of expression profiles of genes that were differentially expressed in NB4-LR1 compared to NB4-LR1^SFD^ ATRA-treated cells. Red indicates upregulated genes and green indicates downregulated genes **d** Venn diagrams showing the number of significantly differentially expressed genes (up-regulated and down-regulated) at 7 days of ATRA treatment (1 μM) in NB4-LR1 and NB4-LR1^SFD^. Figures in the overlapping sections indicate the number of differentially expressed genes common to multiple pair-wise cell lines comparison. *H19* is the only gene to be up-regulated in NB4-LR1 ATRA-treated cells but down-regulated in the NB4-LR1^SFD^ cells after ATRA treatment. Lists of corresponding genes are presented in Additional file 2 (Table S2)

A transcriptomic analysis on microarray was performed on the two ATRA-induced maturation-resistant NB4-LR1 and NB4-LR1^SFD^ cell lines to identify genes differentially expressed upon ATRA treatment. We isolated total RNAs from ATRA-treated or non-treated cells NB4-LR1 and NB4-LR1^SFD^ cells. Day 7 was chosen because, at this time of treatment, the *hTERT* expression level was reduced in NB4-LR1 cells (to about 90% of the non-treated cells), whereas it was stabilized in NB4-LR1^SFD^ cells (at about 60% of the non-treated cells) (Fig. 1a).

Transcriptome profiling identified in NB4-LR1 cells, 256 genes that were differentially expressed between ATRA-treated and non-treated cells. Among these, 183 were upregulated and 73 were downregulated. In NB4-LR1^SFD^ cells, only 65 genes were differentially expressed between treated and non-treated cells. Among them, 25 were downregulated whereas 40 were upregulated (Additional file 2: Table S2). The differentially expressed genes were visualized in the form of a volcano plot (Fig. 1b). The expression of the significant genes was displayed in a heatmap (Fig. 1c). Venn diagram representation (Fig. 1d) showing the overlap between genes up- or down-regulated in NB4-LR1 treated vs control cells and NB4-LR1^SFD^ treated vs control cells enabled us to focus our attention on *H19* gene, coding a long non-coding RNA, because it is the only gene whose expression was induced after ATRA treatment in NB4-LR1 cells but repressed in NB4-LR1^SFD^ cells exposed to the same treatment.

**Fig. 2:**
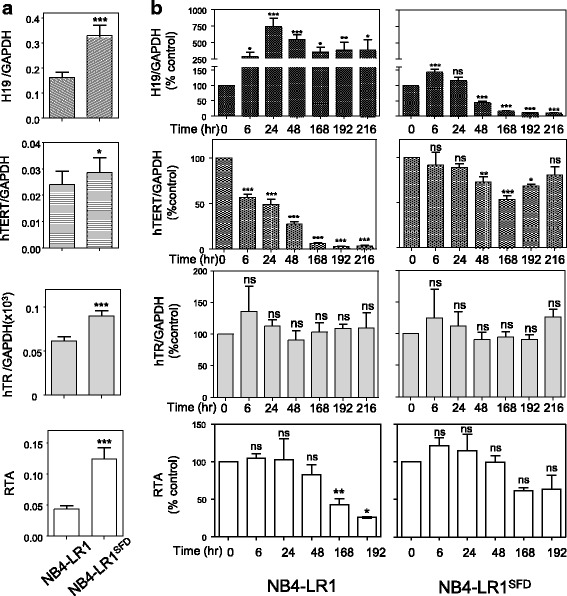
*H19* is induced upon ATRA treatment in NB4-LR1 cells in association with *hTERT* repression, whereas it is repressed in the NB4-LR1^SFD^ cells resistant to ATRA-induced *hTERT* repression. **a** Basal expression levels of *H19*, *hTR*, and *hTERT* mRNA and relative telomere activity (RTA) in NB4-LR1 and NB4-LR1^SFD^ cells. **b** Expression levels of *H19*, *hTR*, and *hTERT* mRNA and RTA after treatment of NB4-LR1 and NB4-LR1^SFD^ cells with ATRA (1 μM) for the indicated time. All experiments were repeated at least 3 times. Expression levels were normalized to GAPDH expression. RTA measured by qTRAP as described in “Methods” is expressed as the percentage of that detected in the untreated cells. Results were expressed as means +/− SEM. t-test (**a**) or one way ANOVA with post-hoc Tukey, ns *p* > 0.05, *p < 0.05, **p < 0.001, ***p < 0.001

To validate this observation, qRT-PCR was performed to investigate *H19* expressions in NB4-LR1 and NB4-LR1^SFD^ cells treated with ATRA (1 μM) for different times. Figure 2a showed that the constitutive H19 expression is higher in NB4-LR1^SFD^ than in NB4-LR1 cells. The expression of *hTR* and *hTERT* RNA were also higher in NB4-LR1^SFD^ compared to NB4-LR1 cells, which is consistent with a difference in telomerase activity. In NB4-LR1 cells, ATRA treatment induced a strong increase in the expression of *H19* associated with a progressive decrease of *hTERT* expression. In contrast, in the NB4-LR1^SFD^ cell line, as already reported in Fig. 1a, despite the continuous presence of ATRA, a high level of *hTERT* mRNA (more than 50% of expression compared to the untreated cells) of control expression) and telomerase activity was maintained. This partial resistance to ATRA-induced *hTERT* repression was associated with an important decrease of *H19* expression after a slight but significant increase (Fig. 2b). Note that *hTR* expression level was not modified upon ATRA treatment in both cell lines.

**Fig. 3:**
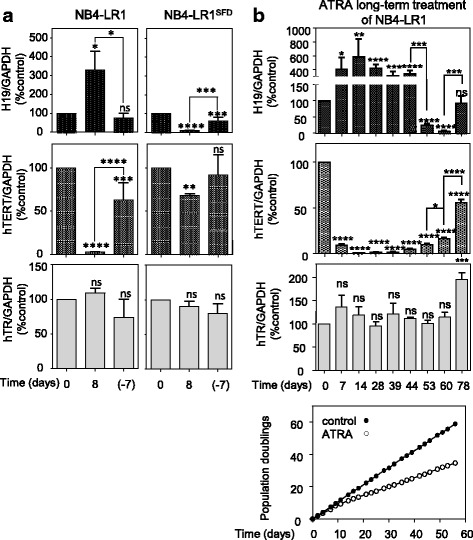
**a** The effects of ATRA treatment on *H19* expression are reversible in both NB4-LR1 and NB4-LR1^SFD^. After culturing cells for 8 days in the presence of ATRA (1 μM), the NB4-LR1 cell culture was switched to ATRA-free medium. (− 7) refers to the NB4-LR1 cells that were cultured 7 days after ATRA removal. Expression levels of *H19*, *hTERT* mRNA, and *hTR* were quantified and normalized to GAPDH expression. Results were expressed as means +/− SEM. One way ANOVA with post-hoc Tukey, ns *p* > 0.05, *p < 0.05, ***p < 0.001, ****p < 0.0001. **b** Establishment of an NB4-LR1^SFD^ like cell line from NB4-LR1 cell line cultured in the continuous presence of ATRA (1 μM). By day 50, in several experiments, most of the cells underwent cell death. However, in two separate experiments a new population, similar to the NB4-LR1^SFD^, overcame this cell death step. The lower panel shows the population doublings of one of the two cultures. At the indicated times, RNA was extracted and *hTERT*, *H19,* and *hTR* expressions measured. Expression levels were normalized to *GAPDH* expression. Note from day 53 the decrease of *H19* expression (upper panel) associated with *hTERT* re-expression (middle panel) t-test or one way ANOVA with post-hoc Tukey, ns *p* > 0.05, *p < 0.05, ***p < 0.001. ****p < 0.0001

We have previously shown that *hTERT* repression by prolonged ATRA treatment was reversible [5]. Similarly, in both NB4-LR1 and NB4-LR1^SFD^ cells, the effects of ATRA treatments on *H19* expression were reversible (Fig. 3a). Indeed, the withdrawal of ATRA from cell cultures at day 8 induced a complete (NB4-LR1) or partial return (NB4-LR1^SFD^) of *H19* expression to the baseline level before ATRA treatment. In parallel, *hTERT* expression is nearly (NB4-LR1) or completely restored (NB4-LR1^SFD^) in cells after ATRA removal. It is worthy to mention, as previously noticed, that *hTR* expression level remained unchanged as compared with the control cells throughout the experiment.

The NB4-LR1^SFD^ cell line was generated after a prolonged culture of NB4-LR1 cells under the selective presence of ATRA [7]. This cell line that has been established as a permanent cell line, overcomes the death induced through a telomere-dependent pathway, proliferates, and expresses high levels of telomerase despite continuous ATRA treatment (Figs. 1a and 2b). We performed a new similar experiment on NB4-LR1 cells in order to investigate *H19* expression during the procedure of selection of resistant cells to ATRA-induced *hTERT* repression. As previously observed [7], many attempts failed and cells died upon ATRA prolonged exposure. However, in two separate experiments, a population of cells emerged from the culture by day 50. Examination of *hTERT* and *H19* expressions revealed that the high repression of *hTERT* during the first 5 weeks of treatment was associated with a high increase of *H19* expression (Fig. 3b). However, around day 50, when most of the cells progressed toward the death we observed a rapid decrease of *H19* expression and the re-expression of *hTERT* associated with recovered cell growth despite the continuous presence of ATRA.

H19 encodes two conserved miRNAs processed from its first exon, miR-676-5p and miR-675-3p [9]. Using several in silico bioinformatics web-based analysis including TargetScan (URL:http://www.targetscan.org), RNA22 (URL: https://cm.jefferson.edu/rna22), and miRanda (URL:https://omictools.com/miranda-tool), we found that hTERT mRNA was predicted as a potential, target of miR-675-5p, although poorly conserved. Therefore, we investigated the expression of this miRNA in both cell lines before and after ATRA treatment (Additional file 3: Figure S1a and b). The expression of miR-675-5p did not exhibit any significant difference either before or after ATRA treatment of NB4-LR1 and NB4-LR1^SFD^. Of note only a significant increase of miR-675-3p expression was observed in ATRA-treated NB4-LR1 cells.

### *H19* expression is induced in the NB4-LR1^SFD^ cells by alternative treatments that repress *hTERT* expression and decrease telomerase activity

**Fig. 4:**
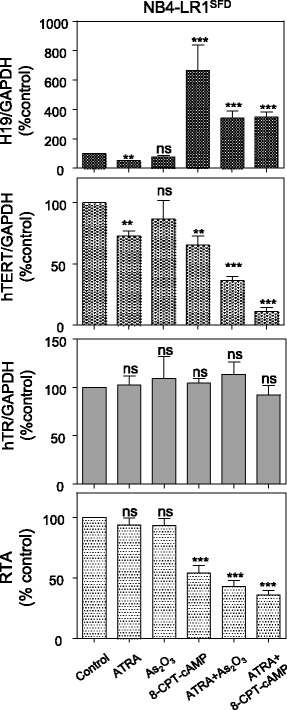
*H19* expression is induced in the NB4-LR1^SFD^ cells by alternative treatments. Expression levels of *H19, hTERT*, and hTR and telomerase activity after treatment of NB4-LR1^SFD^ cells for 48 h with ATRA (1 μM) alone or in combination with either 8-CPT-cAMP (100 μM) or Arsenic trioxide (As_2_O_3_, 0.2 μM).. Expression levels were normalized to *GAPDH* expression. Results were expressed as means +/− SEM. t-test or one way ANOVA with post-hoc Tukey, *p < 0.05, **p < 0.01, ***p < 0.001, ****p < 0.0001

In contrast with the parental NB4 cells that are sensitive to ATRA-induced maturation, both NB4-LR1 and NB4-LR1^SFD^ cell variants fail to mature upon ATRA treatment alone. However, maturation can be rapidly induced following the cooperative stimulation by ATRA and cAMP analog [7, 10]. This maturation has been associated with *hTERT* repression and a decrease of telomerase activity. Therefore, the expression of *H19* was measured in NB4-LR1^SFD^ cells during the induction of maturation by the combination of ATRA and the cAMP analog, 8-CPT-cAMP. As shown in Fig. 4a, this combination induced in the NB4-LR1^SFD^ cells an increase of *H19* expression associated with a maturation-dependent repression of *hTERT*. Of note, 8-CPT-cAMP treatment alone induced a strong increase of *H19* expression. This increase was accompanied with a reduction of *hTERT* mRNA level similar to that seen after ATRA treatment (about 30%). However, 8-CPT-cAMP treatment of these cells induced also a reduction of telomerase activity not observed after ATRA treatment. This suggests that the decrease of telomerase activity cannot be explained by *hTERT* repression alone. Considering that arsenic trioxide (As_2_O_3_) combined with ATRA is also able to repress *hTERT* in the NB4-LR1^SFD^ cells [11], we conducted an experiment to determine whether this combination affects also *H19* expression. As previously shown, we observed that, in NB4-LR1^SFD^ cells, this combined treatment induced a repression of *hTERT* (Fig. 4b). Moreover, this repression was associated with an increased *H19* expression. The treatment with As_2_O_3_ alone changed neither expressions of *H19*, *hTERT* and *hTR* nor activity of telomerase. Altogether, these observations demonstrate that, in the NB4-LR1^SFD^ cells, different treatments that increase *H19* expression lead to a decrease of telomerase activity associated to a certain extent with *hTERT* gene repression.

### An inverse correlation between the expressions of *hTERT* and *H19* is observed in APL patients

**Fig. 5:**
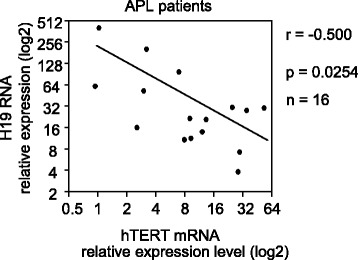
Negative correlation between the expression of *hTERT* and *H19* in acute promyelocytic leukemia patients. The correlation analysis of *H19* and *hTERT* expression was performed using the Pearson correlation test with the software of GraphPad Prism 6.01

Given the above results demonstrating, in the APL cellular model, an inverse correlation between *H19* and *hTERT* expression levels, we wondered whether this correlation could be found also in APL patient cells. Therefore, we analyzed the expression levels of *hTERT* and *H19* using publicly accessible expression profiles of 16 APL patients (TCGA, http://cbioportal.org). As indicated in Fig. 5, there was a negative correlation between *H19* and *hTERT* expressions in these patients (*r* = − 0.500, *p* < 0.05). This observation reinforces the idea that there is a link between *H19* and *hTERT*.

### *H19* LncRNA inhibits telomerase activity

**Fig. 6:**
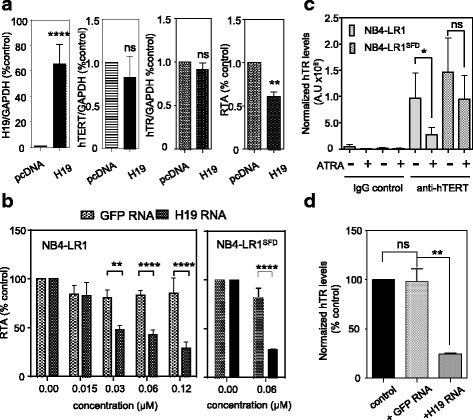
*H19* RNA inhibits telomerase activity by altering the assembled telomerase complex. **a** Influence of *H19* overexpression on *hTR* and *hTERT* expression and telomerase activity. NB4 cells were submitted to nucleofection in the presence of H19 (H19-pcDNA) or empty vector (pcDNA). Six hours after nucleofection, proteins and RNA were extracted. The levels of *H19, hTR* RNA, and *hTERT* mRNA were quantified by qRT-PCR and normalized to the levels of *GAPDH* mRNA. RTA was measured by qTRAP. **b** In vitro concentration-dependent inhibition of telomerase activity by *H19*. NB4-LR1 cells were extracted with CHAPS buffer. In vitro transcribed *H19* was incubated with protein extracts for 90 min. Before assessment of telomerase activity. In vitro transcribed *GFP* was included as a specificity control. RTA measured by qTRAP is expressed as the percentage of that detected in protein extracts not supplemented with RNA molecules. **c** Lysates prepared from NB4-LR1 and NB4-LR1^SFD^ cells treated or not with ATRA (1 μM) for 48 h were subjected to immunoprecipitation (IP) with IgG or anti-hTERT antibodies. The immunoprecipitates were extracted for *hTR* analysis by quantitative RT-PCR. **d** Lysates from non-treated NB4-LR1 cells were preincubated with in vitro transcribed *H19* RNA molecules subjected to IP using the hTERT antibody**.** The immunoprecipitates were then incubated for 90 min. Co-precipitated RNA were extracted and *hTR* assembled with hTERT quantified by qRT-PCR. As a control, an in vitro transcribed *GFP* RNA was used. Results were expressed as means +/− SEM. t-test or two way ANOVA *p < 0.05, **p < 0.01, ***p < 0.001, ****p < 0.0001

To examine an effect of *H19* on *hTERT* expression or telomerase activity independently of ATRA treatment, we performed a transient overexpression of *H19*. This experiment was performed on the parental NB4 cells since in these cells transfection is much more efficient and much less toxic than in NB4-LR1 and NB4-LR1^SFD^ cell sublines. The results in Fig. 6a indicate that *H19* overexpression in NB4 cells led to a significant decrease of telomerase activity measured by TRAP assay compared to NB4 cells transfected with empty pcDNA 3.1(+) vector, whereas no significant change in neither *hTERT* mRNA nor in *hTR* levels was observed.

To explore further the interfering effect of *H19* on telomerase activity, we performed a qTRAP assay in cell extracts from NB4-LR1 cells incubated with increasing amounts of in vitro transcribed *H19* RNA. Figure 6b shows that incubation with *H19* RNA produced a marked inhibition of the telomerase activity present in cell extracts from NB4-LR1 cells. The inability of the irrelevant *GFP* RNA to inhibit telomerase activity demonstrated this selectivity. The inhibitory effect of *H19* on telomerase activity is also observed when incubation was performed using NB4-LR1^SFD^ cell lysates. Taken together, these data indicate that *H19* transcribed in vivo or in vitro could inhibit telomerase activity.

### *H19* promotes telomerase inhibition by affecting the assembled telomerase complex

Human telomerase is an RNA binding protein that consists at least of two core subunits, hTERT protein and a template RNA subunit (*hTR*). Both hTERT and *hTR* are essential for the assembly of a functional active telomerase in vitro and in vivo [12]. Therefore, we hypothesized that one possible mechanism by which *H19* can inhibit telomerase activity might be through its influence on the proper telomerase assembly into a functional complex and might involve *hTR*. To explore this assumption, we performed RNA immunoprecipitations (RIP) on lysates from NB4-LR1 or NB4-LR1^SFD^ cells treated or not with ATRA (1 μM) for 48 h with an anti-hTERT antibody or a normal rabbit IgG, and measured by qRT-PCR the amount of hTR in the pellet fraction of the immunoprecipitation reactions. The antibody against hTERT has already been previously validated [13] and was further verified (Additional file 4: Figure S2). Only the *hTR* bound to hTERT can be recovered in the immune complex. Figure 6c shows that *hTR* level was lower in the immunoprecipitates from NB4-LR1 cells treated with ATRA relative to non-treated NB4-LR1 cells. To further investigate a direct role of *H19* on telomerase activity a complementary experiment was performed using lysates from NB4-LR1 cells supplemented with either the *H19* RNA or an irrelevant *GFP* RNA, and then subjected to immunoprecipitation. Figure 6d shows that the lysate supplementation with *H19* decreased the amount of *hTR* immunoprecipitated with hTERT. As a control for specificity, we added the irrelevant GFP RNA in cell lysate and showed no significant changes in the amount of *hTR* in the hTERT immunoprecipitate.

## Discussion

In this study, using a pair of well-established cell lines that respond differently to ATRA treatment in terms of telomerase expression, we identified a new pathway through which ATRA can regulate telomerase activity. This new pathway involves *H19*, a long non-coding RNA.

We have previously shown that in the maturation resistant NB4-LR1 APL cells, ATRA induced a strong transcriptional repression of *hTERT* gene expression in the absence of cell differentiation. In the present study, we demonstrated that in parallel, ATRA induces a marked increase of H19 expression. In contrast, in the NB4-LR1^SFD^ cell line, which in addition is resistant to ATRA-induced repression of *hTERT*, ATRA treatment induced a repression of *H19*. Importantly, treatments of these cells with ATRA combined to either cAMP analogs (to activate PKA signaling pathway and induce differentiation) or arsenic trioxide induced *H19*. In both cases of treatments, the increase of *H19* levels occurs in parallel with the repression of *hTERT* suggesting an inverse relationship between *H19* and *hTERT* expression at the level of the RNA. The precise events downstream of ATRA that result in *H19* induction are not known and requires further investigation. An induction of *H19* transcription by ATRA has already been described in cells derived from human testicular germ tumors associated with growth inhibition [14]. Notably, exploring the AML patient cohort from TCGA atlas, we found the same inverse relationship in the subgroup of APL patient. Even though this subgroup included only 16 patients, taken together these data strongly suggest an inverse relationship between the expression of *hTERT* and *H19*.

Long non-coding RNAs (LncRNAs) have recently emerged as a novel group of non-coding RNAs able to regulate mammalian gene expression. Besides their participation in normal physiology, LncRNA have been implicated in cancer development and progression [15, 16].

*H19*, an oncofetal gene that does not code for a protein, is transcribed to a non-coding RNA. It is located on human chromosome 11p15.5 within the highly conserved imprinted H19/insulin-like growth factor 2 (IGF2) locus. These two genes are reciprocally imprinted, leading to differential allelic expression of *H19* from the maternal allele and *IGF2* from the paternal allele [17]. Although *H19* has been intensively studied in genomic imprinting, the function of H19 as a non-coding RNA has only recently begun to be investigated [18]. *H19* was initially identified as a tumor suppressor. Indeed, its overexpression in some tumor cells was associated with inhibition of proliferation, morphological changes, decrease of clonogenicity in soft agar, and tumorigenicity in nude mice [19]. Furthermore, downregulation of *H19* gene expression was recognized as an early event in the formation of several tumor types [20–22]. However, increased expression of *H19* has also been observed in a variety of tumors, suggesting that *H19* is, in contrast, essential for tumor growth [23–29]. A link has been well established between *H19* and several tumorigenesis related genes, such as c-Myc and E2F1 [30, 31]. Thus, it remains controversial as to whether *H19* functions as a tumor promoter or a tumor suppressor. It is possible that *H19* plays differential roles depending on the developmental stage and/or the tissue type. The investigations on *H19* functions were more widely depicted in solid tumors than in leukemia. In addition, the exact mechanism by which *H19* as a non-coding RNA functions remain to be elucidated. Long non-coding RNA, including H19 can directly modulate the transcription of its target genes either by recruitment of chromatin modifiers or sequestration of regulatory proteins or regulate mRNA degradation and translation [32–36]. We have previously shown that epigenetic modifications targeting the distal region of hTERT promoter could account for the capacity of retinoids to repress hTERT [37]. However, investigations using invalidation and overexpression experiments failed to reveal any direct effect of *H19* on *hTERT* expression, which made it unlikely that *H19* could have a direct effect on hTERT expression independently of ATRA treatment.

*H19* as a primary miRNA transcript generates two mature miRNAs, miR-675-3p and miR-675-5p [9]. These two conserved miRNAs play important roles in the regulation of *H19*-mediated processes. *H1*9 could, therefore, at least in part, function through these miRNAs. Using bioinformatics miRNA target prediction software indicated *hTERT* mRNA as a potential, although poorly conserved, target of miR-675-5p. However, no significant change of the expression of miR-675-5p was observed during ATRA treatment of NB4-LR1 cells, which makes it unlikely a direct action of this miRNA on *hTERT* mRNA level.

Many LncRNA, including *H19*, participate in molecular regulation pathways through their interactions with proteins and modulation of their activities [38]. Telomerase enzyme is unusual: it has a protein component that has an affinity for *hTR*, its natural RNA partner essential for its activity. Using an in vitro telomerase assay, we observed that the addition of in vitro transcribed *H19* RNA molecules to protein extracts prepared from either NB4-LR1 or NB4-LR1^SFD^ cells inhibited telomerase activity. Furthermore, we demonstrated that the ectopic expression of *H19* in NB4 cells induced a decrease of telomerase activity without any modifications of *hTERT* expression indicating that the mechanism of action might involve *hTR*. This hypothesis was further supported by the observation that 8-CPT-cAMP treatment can induce alone an important increase of *H19* associated with a notable loss of telomerase activity whereas *hTERT* expression remained relatively high. Using RIP experiments, we showed that *H19* decreases the amount of *hTR* associated with hTERT component and thereby impairs the functions of this enzyme. We were not able to demonstrate a direct interaction of *H19* with either hTERT protein or its RNA component *hTR*. Indeed, the amount of hTERT in a cell is very low [39] and western blot are not sensitive enough to detect the endogenous protein. It is likely that *H19* does not bind tightly the telomerase ribonucleoprotein but instead is recruited either transitorily or through another telomerase auxiliary factor. The interaction of hTERT with another RNA component different from *hTR* has already been reported. Indeed, using a tandem affinity peptide-tagged hTERT protein in Hela S3 cells, Maida et al. identified a heterogeneous mixture of 38 RNA sequences associated with hTERT, including the RNA component of mitochondrial RNA processing endoribonuclease (RMRP) [40]. TERT has also been reported to interact with mitochondrial tRNAs and amino-acylo tRNA synthases [41]. Another possible mechanism for the decrease of *hTR* binding to hTERT protein is the *H19* interference with free *hTR* preventing it from associating with hTERT.

Our results contrast with those previously published [42] showing that overexpression of *H19* enhances the binding of hTERT to *hTR* and leads to an increase of telomerase activity. In this study, Pu et al. provided no information regarding the hTERT antibody used in the RIP experiment performed. However, the lack of specificity of most commercially available antibodies directed against the hTERT protein is well-known within the scientific community in this field [13]. The specificity of the antibody used here has already been well characterized [13] and further verified in the present work. Another explanation for such discrepancy is the difference in the cellular model used in their study (liver cancer stem cells). One possibility is that, *H19* could be a molecular chaperone able to promote either the association or the dissociation of hTERT to *hTR*, depending on the cellular context.

**Fig. 7:**
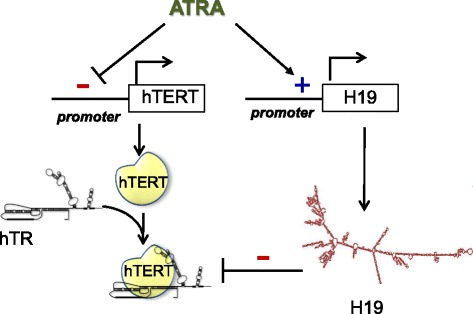
Hypothetic schematic overview of the relationship between *H19* and hTERT in ATRA treated APL cells. ATRA might target telomerase through two distinct processes to ensure a sustained inhibition of telomerase activity, *first* by decreasing *hTERT* gene transcription and consequently the amount of hTERT protein, and second, by increasing *H19* level, which in turn may impede telomerase assembly and functions

Our results have broader implications considering the antitelomerase action of retinoids and thereby their antitumor properties independently of their effects on differentiation: we propose that ATRA might target telomerase through two distinct processes to ensure a sustained inhibition of telomerase activity, *first* by decreasing *hTERT* gene transcription and consequently the amount of hTERT protein, and *second*, by increasing *H19* level, which in turn may impede telomerase assembly and functions (Fig. 7). Further studies are required to fully elucidate the precise mechanisms by which *H19* interfere with the activity of the telomerase complex.

## Conclusions

In conclusion, this study identifies for the first time a new level of telomerase regulation through the involvement of *H19,* and thereby reveals also a novel function for this LncRNA in telomerase biology. It also suggests that directly targeting hTERT/*hTR* complex through an increase of *H19* might provide a new treatment strategy for cancer that could have clinical utility.

## Additional files

Additional file 1: **Table S1.** Primer sequences. (DOCX 21 kb)

Additional file 2: **Table S2.** Data analysis of the microarray experiments. Lists of genes with significant differential expression levels (fold changes of ±2 fold; *p* < 0.01). Some details correspond to the results displayed in Venn diagrams presented in Fig. 1c of the main text. (XLSX 66 kb)

Additional file 3: **Figure S1.** Expression levels of mir-675-5p and mir-675-3p in non-treated (a) and after 1 μM ATRA treatment (b) of NB4-LR1 and NB4-LR1^SFD^ cells. Expression levels were normalized to SNORD44 expression. Results were expressed as means +/− SEM. One way ANOVA with post-hoc Tukey, ns *p* > 0.05, *p < 0.05, ***p < 0.001. (PDF 103 kb)

Additional file 4: **Figure S2.** Validation of the anti-hTERT antibody specificity. Immunoprecipitation of the hTERT/hTR complex was performed using an anti-hTERT antibody (Rockland) or pre-immune IgG as described in Material and Methods. The presence of *hTR* was detected by quantitative RT-PCR. Results were expressed as means +/− SEM. t-test *p < 0.05. (PDF 90 kb)

## Abbreviations

APL: Acute promyelocytic leukemia
ATRA: All-*trans* retinoic acid
GAPDH: Glyceraldehyde-3-phosphate dehydrogenase
GFP: Green fluorescent protein
hTERT: Human telomerase reverse transcriptase
hTR: Human telomerase RNA
IP: Immunoprecipitation
LncRNA: Long non-coding RNA
qRT-PCR: Quantitative reverse transcriptase polymerase chain reaction
qTRAP: Quantitative telomeric repeats amplification protocol
