# Supplementary material for: Telomerase regulation by the long non-coding RNA H19 in human acute promyelocytic leukemia cells
Source: Mol Cancer. 2018 Apr 27;17:85. doi: 10.1186/s12943-018-0835-8 (PMC5923027; doi:10.1186/s12943-018-0835-8)
Supplement: Supplementary file 1 — Table S1. Primer sequences. (DOCX 21 kb) [file 12943_2018_835_MOESM1_ESM.docx]

**Telomerase regulation by the long non-coding RNA H19 in human acute promyelocytic leukemia cells**

Joelle EL HAJJ^1,2,3,4^, Eric NGUYEN^1,2^, Qinyuan LIU^1,2,#^, Claire BOUYER^1,2^, Eric ADRIAENSSENS^5^, George HILAL^4^, Evelyne SÉGAL-BENDIRDJIAN^1,2,3^.

^1^ INSERM UMR-S 1007, Cellular Homeostasis and Cancer, Paris, France

^2^ Paris-Descartes University, Paris Sorbonne Cité, Paris, France

^3^ Paris-Sud University, Paris-Saclay University, Orsay, France

^4^ Cancer and Metabolism Laboratory, Faculty of Medicine, Saint-Joseph University, Beirut, Lebanon

^5^ INSERM U 908, University Lille 1, Villeneuve d’Ascq, France

^#^ Present address: Bristol-Myers Squibb (China) Investment Co., Ltd. Shanghai 200040, P.R.China

**Additional files**

**Additional file 1: Table S1:** **Primer sequences**.

| Primer name | qRT-PCR primer sequence (5' to 3') |
| --- | --- |
| hTERT | FW : CGGAAGAGTGTCTGGAGCAA  RV : CTCCCACGACGTAGTCCATG |
| H19 | FW : TGCTGCACTTTACAACCACTG |
|  | RV : ATGGTGTCTTTGATGTTGGGC |
| GAPDH | FW:CACCCATGGCAAATTCCATGGC |
|  | RV:GCATTGCTGATGATCTTGAGGCT |
| hTR | FW : CCTAACTGAGAAGGGCGTAGGC  RV : CTAGAATGAACGGTGGAAGGCG |
| hsa-miR-675-5p | GAGAGGGCCCACAGTGAAAA |
| hsa-miR-675-3p | GTATGCCCTCACCGCTCAA |
| SNORD44 | GCAAATGCTGACTGAACATGAA |
|  | qTRAP assay primer sequence (5’ to 3’) |
| TS | AATCCGTCGAGCAGAGTT |
| ACX | GCGCGGCTTACCCTTACCCTTACCCTAACC |
