# Supplementary figures and images for: Telomerase regulation by the long non-coding RNA H19 in human acute promyelocytic leukemia cells
Source: Mol Cancer. 2018 Apr 27;17:85. doi: 10.1186/s12943-018-0835-8 (PMC5923027; doi:10.1186/s12943-018-0835-8)

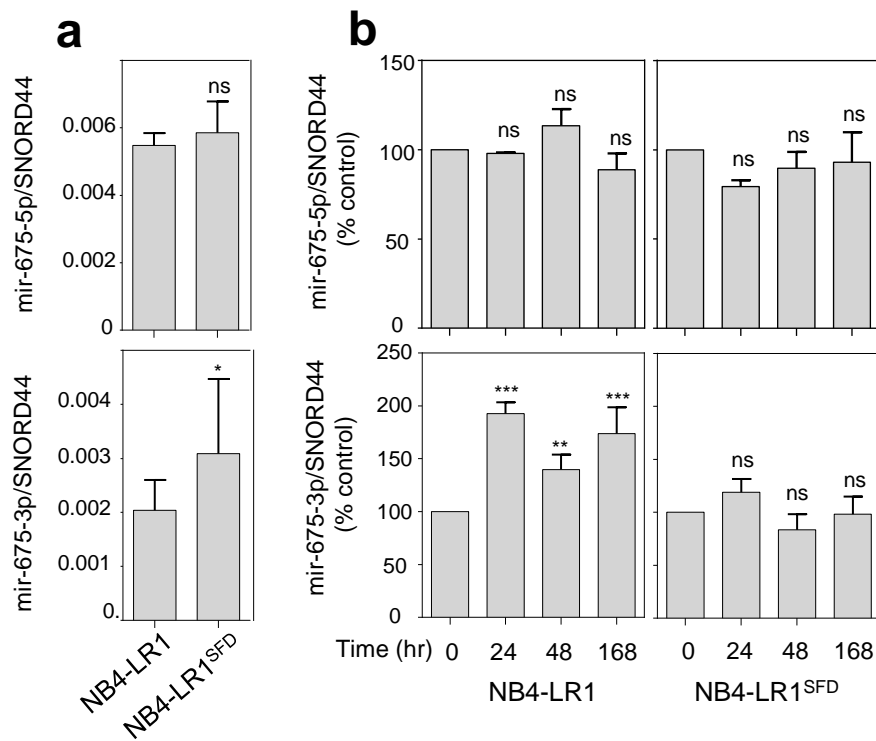

Supplement: Supplementary file 3 — Figure S1. Expression levels of mir-675-5p and mir-675-3p in non-treated (a) and after 1 μM ATRA treatment (b) of NB4-LR1 and NB4-LR1SFD cells. Expression levels were normalized to SNORD44 expression. Results were expressed as means +/− SEM. One way ANOVA with post-hoc Tukey, ns p > 0.05, *p < 0.05, ***p < 0.001. (PDF 103 kb) [file 12943_2018_835_MOESM3_ESM.pdf]

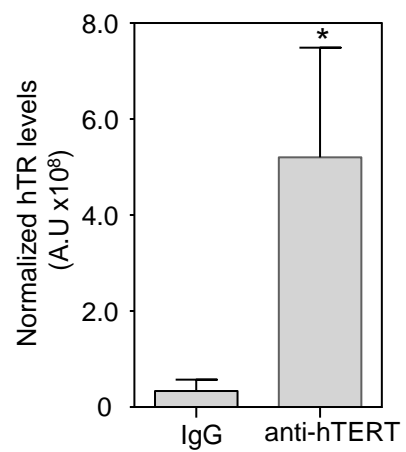

Supplement: Supplementary file 4 — Figure S2. Validation of the anti-hTERT antibody specificity. Immunoprecipitation of the hTERT/hTR complex was performed using an anti-hTERT antibody (Rockland) or pre-immune IgG as described in Material and Methods. The presence of hTR was detected by quantitative RT-PCR. Results were expressed as means +/− SEM. t-test *p < 0.05. (PDF 90 kb) [file 12943_2018_835_MOESM4_ESM.pdf]
